# Diagnostic challenges in cutaneous leishmaniasis due to atypical *Leishmania infantum*: pathologists’ insights from re-emergence zones

**DOI:** 10.3389/fmed.2024.1453211

**Published:** 2024-09-12

**Authors:** Suheyla Ekemen, Muhammed Nalcaci, Seray Toz, Chizu Sanjoba, Cuyan Demirkesen, Emel D. Cetin, Tulay Tecimer, Pelin Yildiz, Mayda Gursel, Umit Ince, Yusuf Ozbel, Cevayir Coban

**Affiliations:** ^1^Vocational School of Health Services, Acibadem University, Istanbul, Türkiye; ^2^Division of Malaria Immunology, Department of Microbiology and Immunology, Institute of Medical Science (IMSUT), The University of Tokyo, Tokyo, Japan; ^3^Japan Science and Technology Research Partnership for Sustainable Development (SATREPS) One Health Project, Tokyo, Japan; ^4^Department of Parasitology, Ege University School of Medicine, Izmir, Türkiye; ^5^Graduate School of Agricultural and Life Sciences, The University of Tokyo, Tokyo, Japan; ^6^Department of Pathology, Acibadem University School of Medicine, Istanbul, Türkiye; ^7^Acibadem Central Pathology Laboratory, Istanbul, Türkiye; ^8^Izmir Biomedicine and Genome Center, Basic and Translational Research Program, Izmir, Türkiye; ^9^International Vaccine Design Center, Institute of Medical Science (IMSUT), The University of Tokyo, Tokyo, Japan; ^10^The University of Tokyo Pandemic Preparedness, Infection and Advanced Research Center (UTOPIA), The University of Tokyo, Tokyo, Japan

**Keywords:** *Leishmania infantum*, cutanaous leishmaniasis, skin tumors, PCR, dermatopathology, surgery, misdiagnosis, reemergence

## Abstract

**Background:**

Leishmaniasis, a parasitic infection affecting both humans and animals, is increasingly spreading across Mediterranean and European regions, largely driven by human migration and environmental changes. In countries like Türkiye and across Europe, which have seen large influxes of migrants, the incidence of cutaneous leishmaniasis (CL) is rising, with cases now appearing in cities where the disease was previously undocumented. In these previously non-endemic areas, physicians unfamiliar with the characteristic lesions may misdiagnose CL, particularly in cases with only cutaneous manifestations. This study aims to evaluate the impact of re-emerging CL on the routine diagnostic practices of pathologists in Türkiye, by retrospectively reviewing cases.

**Methods:**

We conducted a retrospective analysis of CL cases diagnosed between 2013 and 2022 at a single pathology center in Türkiye, covering multiple provinces. Twelve cases of CL were identified and analyzed based on clinical presentation, pre-diagnosis, histopathological findings, and molecular diagnostics. DNA extraction and PCR were performed on paraffin-embedded tissue samples to identify the *Leishmania* species involved.

**Results:**

Out of the twelve CL cases reviewed, seven exhibited morphological findings strongly suggestive of CL (MFSS of CL), warranting further microbiological evaluation. All patients presented with non-healing skin lesions characterized by central ulceration, crater-like formations, or papulonodular lesions. Notably, CL was included in the clinical pre-diagnosis in only 58.3% of cases, while it was not considered in the remaining 41.7% of cases. Clinicians initially pre-diagnosed skin tumors in six cases (50%), four of which led to wide surgical excision. Histopathological examination in all cases revealed chronic or mixed (acute/chronic) inflammation, predominantly rich in histiocytes. To further investigate the role of *Leishmania* species in the pre-diagnosis, DNA extraction and PCR were performed on paraffin-embedded tissue samples, identifying *L. infantum* as the causative agent in 10 cases and *L. major* in two cases. Notably, *L. infantum* was the causative agent in all five cases initially misdiagnosed as skin tumors, which were also associated with a granulomatous type of chronic inflammation.

## Introduction

Leishmaniasis is a parasitic infection caused by the genus *Leishmania*, belonging to the family *Trypanosomatidae*, which includes over 20 species (1). The disease affects both humans and animals, and is transmitted through the bite of an infected phlebotomine female sand flies (2–4). Leishmaniasis is prevalent in approximately 100 countries, putting 350 million people at risk, not only due to its potential for causing fatalities but also due to its significant socioeconomic impact (5–7). An estimated 2 million new cases occur annually, making it the second most deadly parasitic disease after malaria (8–10). The spread of the disease is influenced by climatic, environmental, and socioeconomical factors (7). The disease is strongly associated with poverty, malnutrition, and inadequate housing, and its incidence is rising due to unhealthy living conditions resulting from wars and mass migrations (4, 7). Recent surveys indicate an increased incidence of leishmaniasis in countries such as Türkiye and across Europe, which have experienced large influxes of migrants (4, 11, 12).

Leishmaniasis manifests in three forms based on the distribution of the infection in the body: visceral leishmaniasis (VL, or Kala-azar), which affects internal organs such as the liver, spleen, and bone marrow; cutaneous leishmaniasis (CL), which primarily affects the skin; and mucocutaneous leishmaniasis (MCL), which involves the skin, connective tissue, and cartilage (3, 5, 6). VL is caused by *L. donovani* and *L. infantum* and is more prevalent in immunocompromised individuals (13). The onset of VL is often nonspecific, with early symptoms that mimic other diseases, making diagnosis challenging (14, 15). CL is the most common clinical form of leishmaniasis (5). Unlike MCL and VL, CL is characterized by localized, self-healing skin lesions and is not typically fatal. However, both CL and MCL can be clinically and histopathologically mistaken for skin and soft tissue malignancies (3, 16). Leishmaniasis, in its various forms, can cause severe infections in immunocompromised or immunodeficient individuals, such as transplant recipients, cancer patients, and those with HIV or primary immunodeficiencies (4, 16, 17). CL occurs in all areas where leishmaniasis is endemic. Old World CL, commonly found in the Mediterranean region and the Middle East, is most frequently associated with *L. major, L. tropica* and *L. aethiopica*. However, infections with *L. donovani* and *L. infantum,* typically linked to VL, can also result in solitary cutaneous lesions (13). Painless papules and dermal nodules typically develop within few weeks to several months after a sand fly bite (13, 18, 19). As the lesion progresses, it assumes a crater-like appearance with central ulceration and painless margins, eventually healing with scar tissue formation (13).

The gold standard for diagnosing CL is the identification of 2–4 micrometer-sized amastigotes in Giemsa-stained smears from the lesions by light microscopy (5, 13, 19, 20). However, morphological examination alone is insufficient for species identification (19). Determining the specific *Leishmania* species is crucial for prognosis, control, and treatment, as not all species respond to existing therapies (21). Species identification can be accurately achieved through nucleic acid amplification tests, particularly PCR, which offers high sensitivity and specificity (22, 23).

Leishmaniasis, particularly CL caused by *L. tropica,* has historically been present in Türkiye and surrounding regions for centuries. Prior to 1950s, intensive malaria control efforts, which targeted mosquito vectors, also effectively reduced sand fly populations, leading to a significant decline in leishmaniasis cases and confining the disease to a few cities along the Syria-Iraq border (24). This period of indirect leishmaniasis control resulted in a subsequent lack of trained medical professionals, including medical doctors and microbiologists, with experience in diagnosing and treating CL. Between 2013–2016, there was a dramatic increase in leishmaniasis cases, largely due to the influx of Syrian refugees into Türkiye following the civil war in Syria (25). Recent reports indicate that CL is now spreading to cities in Türkiye that have not seen the disease for decades (26). The catastrophic earthquake in Türkiye in February 2023 has further hightened concerns about a potential leishmaniasis outbreak (27). Additionally, the epidemiology of CL appears to be shifting with cases now being caused not only, by the “traditional” *L. tropica*, but also *L. infantum*, *L. major* and *L. donovani* (28, 29). making this disease challenging for the medical professionals. This evolving epidemiology presents new challenges for medical professionals unfamiliar with these atypical *Leishmania* species.

One of the challenges in diagnosing CL is its potential to mimic tumors, which can lead to misdiagnosis or delayed diagnosis, unnecessary surgical procedures, and postponed treatment (17). With the increasing incidence of leishmaniasis in Türkiye, pathologists in major cities like Istanbul are encountering a growing number of cases initially suspected to be tumors during routine clinical pathology evaluations. To prevent incorrect or delayed treatment by physicians unfamiliar with leishmaniasis, it is crucial to include leishmaniasis in the differential diagnosis and to have a thorough understanding of its histopathological features. Here, we discuss the clinical pre-diagnosis, histopathological evaluation, and *Leishmania* species identified in 12 CL cases in Türkiye, providing insights from the pathologist’ perspective.

## Materials and methods

### Study population and ethics approval

The study population consisted of 12 cases diagnosed with CL after pathological evaluation, sent to the one center (Acibadem University Central Pathology Laboratory, Istanbul) in Türkiye from different provinces and locations between 2013 and 2022. Ethical approval of this retrospective study numbered as ATADEK 2023–15/516 was obtained from Acibadem University Faculty of Medicine Ethics Committee on October 6th, 2023. Informed consent was not required due to the retrospective nature of the study.

### Preparation of pathological specimens

The skin biopsy materials were subjected to routine pathological procedures (30). Briefly, biopsy materials were first fixed using a 10% neutral buffered formalin solution. Next, the paraffin blocks were prepared using a Tissue-Tek Vip^®^ 6 AI device (Sakura Finetek Japan Co., Ltd., Tokyo, Japan). Serial sections of 3 μm thickness were made from all blocks and stained with hematoxylin and eosin (H&E) using a Shandon Gemini Stainer. In addition, Giemsa (109,204, Sigma-Aldrich, Merc)-staining in all cases and, Ehrlich-Ziehl-Neelsen (EZN, Norateks), Gomori Methenamine-Silver Nitrate (GMS, HT100A, Sigma-Aldrich, Merc) and Periodic Acid Schiff (PAS, 1.01646, Sigma-Aldrich, Merc) special histochemical stains in some cases were performed. Skin biopsy preparations were evaluated and reported by expert dermatopathologists (CD, EDC, PY) and a hematopathologist (TT).

### Extraction of DNA from the paraffin blocks

The deparaffinization and DNA extraction were performed using the Qiagen DNeasy isolation kit (Cat. No. 69506), with the addition of a xylene step at the beginning of the procedure, as per the kit protocols.

### Detection of *Leishmania* DNA with ITS-1 real-time PCR method

The real-time PCR method targeting the internal transcribed spacer 1 (ITS-1) region between the SSU and 5.8S rRNA genes specific for *Leishmania* was applied using 20–50 ng genomic DNA of samples to determine the melting temperatures (Tm) for each species as described previously (31). The melting analysis was modified to gather data between 470 and 660 nm wavelength to obtain clearer melt peaks. Three positive controls of *L. infantum* (MCAN/TR/12/EP189), *L. tropica* (MHOM/SY/14/EP200) and *L. major* (MHOM/SU/73/5ASKH) were included to obtain standard curves.

### Differentiation of *Leishmania infantum*/*Leishmania donovani* by cysteine protease B (cpb) PCR

Cysteine protease B PCR was performed for the discrimination of *L. infantum*/*L. donovani* to 10 samples detected as *L. infantum* by real time ITS-1 PCR as described previously by Hide and Laure Banuls (32). The PCR amplification products were visualized by Xpert Green DNA Stain direct (GRiSP, Porto, Portugal) fluorescence after electrophoresis in a 1.5% agarose gel at 100 V for 60 min.

## Results

### Clinical features of the patients

We screened pathology reports from the Acibadem University Medical Pathology Laboratory, which received specimens from various locations in Türkiye, for cases of leishmaniasis between 2013 and 2022. Our analysis identified five cases of CL diagnosed based on the presence of microscopically visible *Leishmania* amastigotes, and seven cases categorized as having morphological findings strongly suggestive of CL (MFSS of CL). Among these 12 patients nine were males and three were females, with ages ranging from 25 to 75 years, and a mean age of 45.5 years. Table 1 summarizes the clinical findings, clinical pre-diagnosis, histopathological features, and pathological diagnoses of patients diagnosed with CL or MFSS of CL. All cases presented with a history of non-healing skin lesions. The lesions were primarily located on sun-exposed areas such as the face, arms, and legs. Clinically, nine cases presented with crater-like lesions featuring ulcerated centers, while three cases exhibited papulonodular lesions. In 11 cases, the lesions were singular; however, one case (case #12) involved multiple nodular lesions that initially appeared on the forehead and later spread to the arms and legs. The clinical pre-diagnoses of these cases varied widely, including skin tumors such as squamous cell carcinoma (SCC) (cases #1, 3), basal cell carcinoma (BCC) (cases #1, 3), skin adnexal tumors (SAT) (cases #6, 10, 11), juvenile xanthogranuloma (case #5), dermatofibroma (case #11), keratoacanthoma (#2), dermatoses discoid lupus erythematosus (DLE) (cases #3, 9, 12), and sarcoidosis (cases #9, 12); and skin infections such as skin tuberculosis (cases #4, 7, 9, 12), fungi (case #7), dracunculiasis (case #4), filariasis (case #4), and nocardiosis (case #4). Leishmaniasis was suspected in 7 of 12 cases (58.3%, cases #3, 4, 5, 7, 8, 9, 12), often alongside other pre-diagnoses. Only one case (8.3% of all cases, case #8) had a single clinical pre-diagnosis of CL. Notably, four out of five cases (80%, cases #1, 2, 6, and 10) that were pre-diagnosed solely as skin tumors underwent nearly complete excision of the lesions, two of which were located on the ear and face.

**Table 1:** Clinical findings, clinical prediagnoses, histopathological diagnoses, and PCR results of the cases.

Case	Age (Year diag.)	Sex	Location	Clinical findings	Clinical pre-diagnosis	Histo-pathological features	Histo-pathological diagnosis	*Leishmania spp*. by PCR
1	75 (2022)	F	Bodrum	Ulcerated non-healing wound on the skin of the left ear	SCC, BCC	NGD	CL	*L. infantum*
2	29 (2020)	M	Bodrum	Mid-ulcerated crater-like lesion on the skin of the left ankle	Keratoacanthoma	NGD	CL	*L. infantum*
3	71 (2020)	M	Gaziantep	Wound that has not healed for 2 months in the right malar area	SCC, BCC, CL, DLE	Mixed (acute and chronic) inflammation	MFSS of CL	*L. infantum*
4	35 (2020)	M	Istanbul (spent one year in Brazil, foreigner)	A skin lesion on the leg has been ulcerated for the past 5 months	Dracunculiasis, filariasis, scrofuloderma, nocardiosis, leishmaniasis	NGD	MFSS of CL	*L. infantum*
5	25 (2020)	M	Istanbul (from Lebanon, foreigner)	Wound on the eyelid skin that has not healed for 7 months	Juvenile xanthogranuloma, leishmaniasis	Diffuse chronic histiocyte-rich inflammation in dermis	MFSS of CL	*L. major*
6	43 (2019)	M	Adana	Subcutaneous nodular lesion 2 cm in diameter in the left mandibular area	SAT	GD	MFSS of CL	*L. infantum*
7	25 (2018)	M	Istanbul (from Kuwait, foreigner)	A papulonodular lesion is present on the left elbow with a persistent discharge for one year	Deep mycosis, atypical Tbc, leishmaniasis	Diffuse chronic histiocyte-rich inflammation in dermis	MFSS of CL	*L. infantum*
8	72 (2018)	F	Istanbul	A non-healing ulcer on the leg that has persisted for a year	leishmaniasis	Diffuse chronic histiocyte-rich inflammation in dermis	CL	*L. infantım*
9	59 (2017)	F	Bodrum	A crusty lesion on the skin of the nose with a raised center that has not healed for 4 months	DLE, sarcoidosis, lupus vulgaris, leishmaniasis	Diffuse chronic histiocyte-rich inflammation in dermis	MFSS of CL	*L. infantum*
10	57 (2014)	M	Istanbul	Subcutaneous nodular lesion in forearm	SAT	NGD	CL	*L. infantum*
11	30 (2013)	M	Istanbul	Papular lesion on the skin of the right leg that has not healed for 2 months	SAT, dermatofibroma	Mixed (acute and chronic) inflammation	MFSS of CL	*L. major*
12	25 (2013)	M	Istanbul	Nodular multiple skin lesions starting on the forehead and spreading to the arms and legs	Tbc, DLE, sarcoidosis, leishmaniasis	Mixed (acute and chronic) inflammation	CL	*L. infantum*

SCC: squamous cell carcinoma, BCC: basal cell carcinoma, DLE: discoid lupus erythematosus, Tbc: Tuberculosis, SAT: skin adnexal tumors, CL: cutaneous leishmaniasis, MFSS of CL: Morphological findings strongly suggestive of cutaneous leishmaniasis, NGD: necrotising granulomatous dermatitis, GD: granulomatous dermatitis.

### Histopathological findings

**Figure 1:**
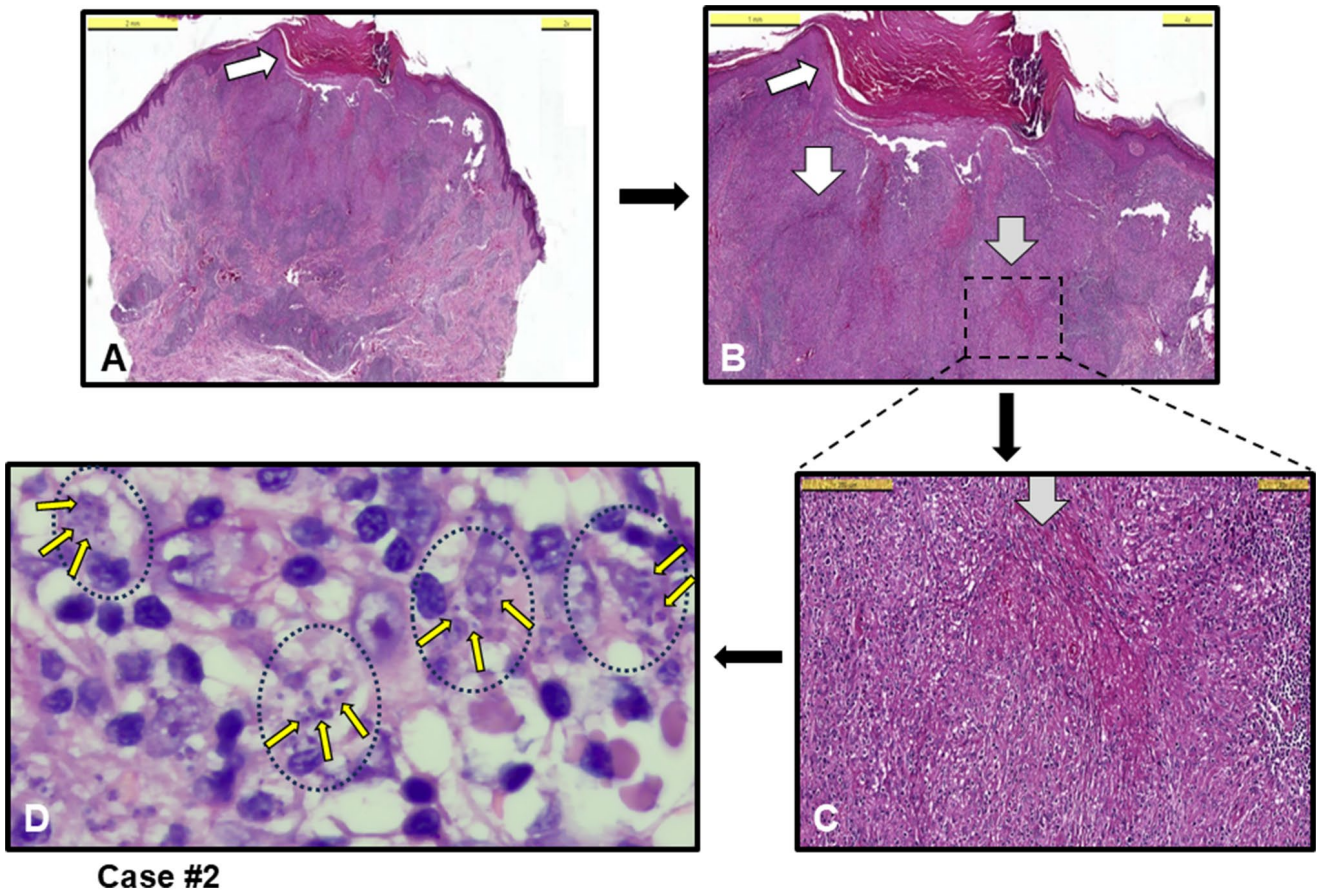
Case #2, a case with necrotizing granulomatous dermatitis (NGD) initially clinically misdiagnosed as keratoacanthoma. **(A,B)** Orthokeratosis and focal parakeratosis (white arrow) in the epidermis, and necrotizing granuloma formations (white arrows), and granuloma structures and intense chronic inflammation (grey arrow) in the dermis [H&E staining, Scale bars 5 mm **(A)** and 1 mm **(B)**]. **(C)** Granuloma structure with dotted-square in **B**, and histiocytic infiltration around grey arrow necrotizing area (H&E, Scale bar 200 μm). **(D)** *Leishmania* amastigotes (example small yellow arrows among many) within histiocytes (example black dotted circles with their nuclei) (Giemsa stain, 100 x magnification with oil). *L. infantum* detected by PCR.

Microscopically, all cases exhibited inflammation characterized by a high density of histiocytes, a morphological terminology used for tissue-resident macrophages. The inflammation was classified as chronic or mixed (acute-chronic). The inflammattory pattern was granulomatous in five cases, with one case (case #6) presenting as non-necrotizing granulomatous dermatitis and four cases (#1, 2, 4 and 10) as necrotizing granulomatous dermatitis (NGD). Figure 1 shows an example necrotizing granuloma structures in the dermis of case #2 (Figures 1A–C), initially misdiagnosed as keratoacanthoma, surrounded by infiltrating histiocytes densely filled with *Leishmania* amastigotes (Figure 1D, Giemsa stained). This case #2 was later found to be infected by *L. infantum* by PCR (Table 1).

**Figure 2:**
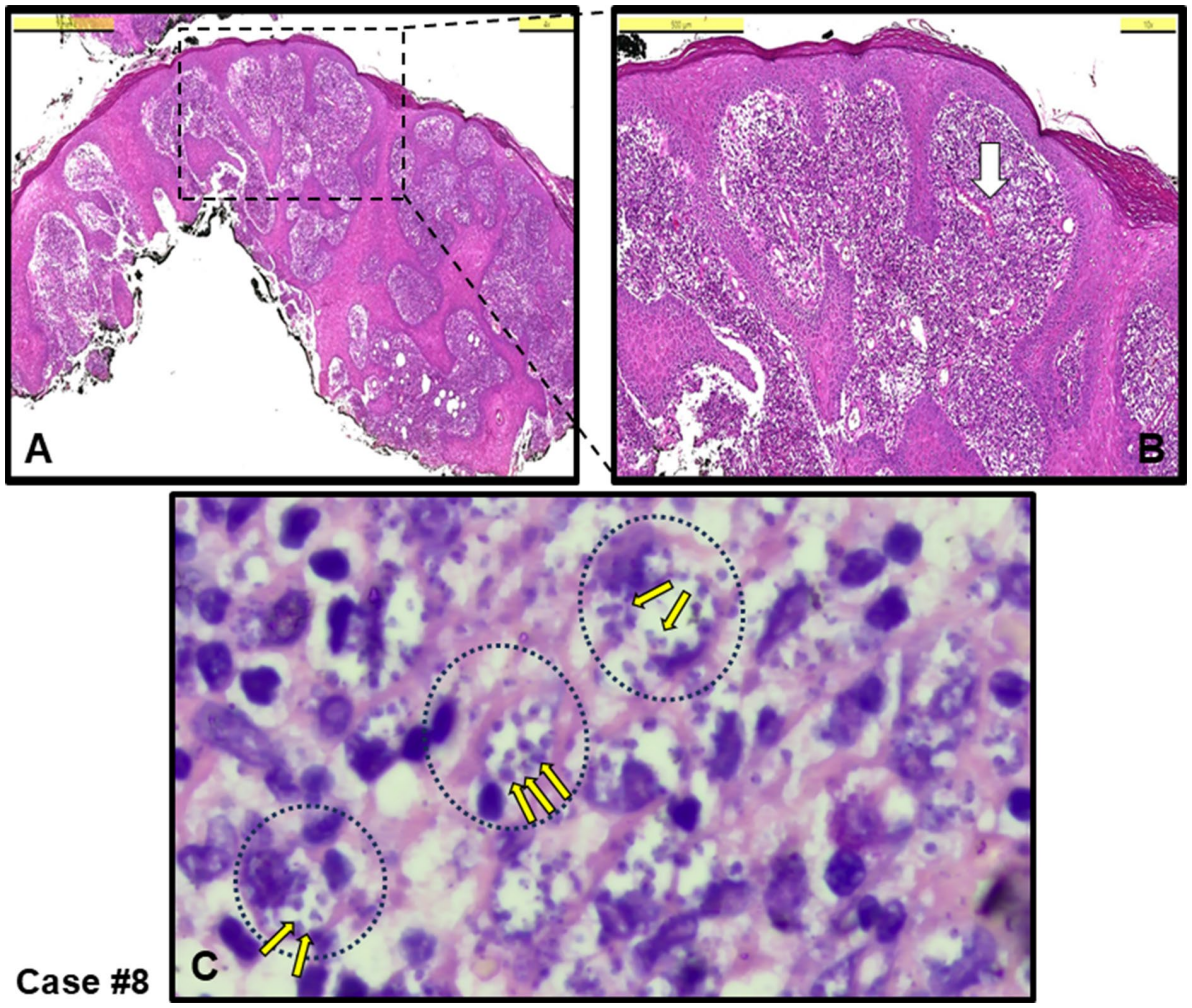
Case #8, a typical case with chronic inflammation, initially clinically pre-diagnosed as leishmaniasis. **(A)** An irregular proliferation in the epidermis, and diffuse infiltration in the dermis rich in histiocytes, including lymphocytes and plasma cells (H&E stain, Scale bar 1 mm). **(B)** Magnified area from the dotted-square in A (H&E stain, Scale bar 500 μm). **(C)** Area shown around white arrow in B. *Leishmania* amastigotes (example small yellow arrows among many) within large histiocytes (example black dotted circles with their nuclei) (Giemsa stain, 100 x magnification with oil). *L. infantum* detected by PCR.

Chronic inflammation, characterized by a diffuse abundance of histiocytes, was observed in four out of the remaining seven cases. Figure 2 shows an example epidermal proliferation and lymphocyte infiltration in the dermis (Figures 2A,B), with large foamy histiocytes densely filled with amastigotes (Figure 2C, Giemsa stained), which was later confirmed by PCR to be infected with *L. infantum.*

**Figure 3:**
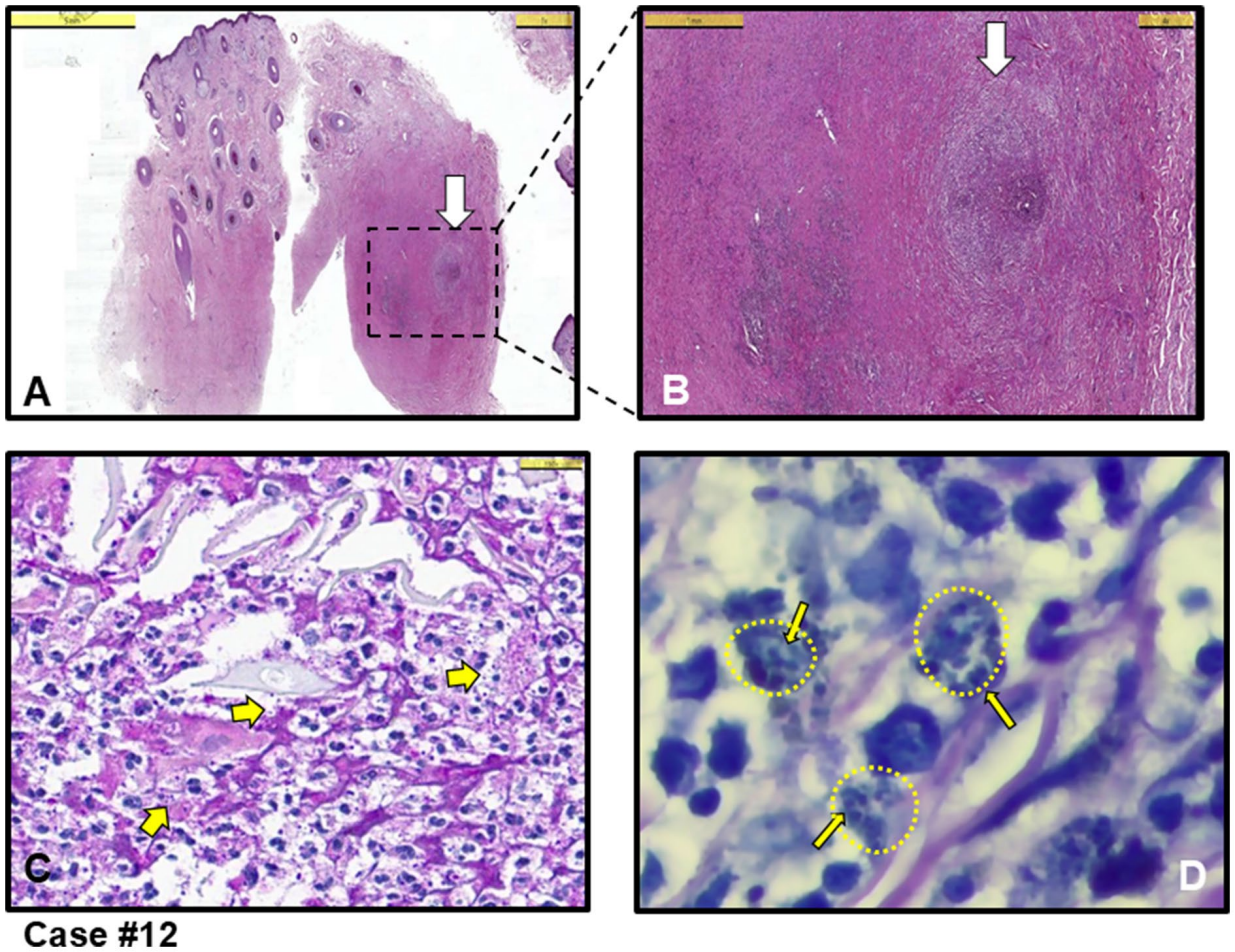
Case #12, a case with mixed (acute and chronic) type inflammation, initially clinically pre-diagnosed with leishmaniasis and other diseases such as Tbc, sarcoidosis and DLE. **(A)** Granulomatous inflammation with necrosis in the dermis (white arrow shows granuloma structure) (H&E stain, Scale bar 5 mm). **(B)** Magnified area in A showing intense inflammation in the dermis and granuloma structure marked with white arrow (H&E stain, Scale bar 1 mm). **(C)** Closer look at the granuloma structure in B. A few thick yellow arrows showing *Leishmania* amastigotes in necrotic areas (H&E stain, Scale bar 25 μm). **(D)** Giemsa staining of the area in C showing clusters of *Leishmania* amastigotes (example thin yellow arrows among many, residing in clusters shown as small yellow-dotted circles) within histiocytes (Giemsa stain, magnified with oil). *L. infantum* detected by PCR.

**Figure 4:**
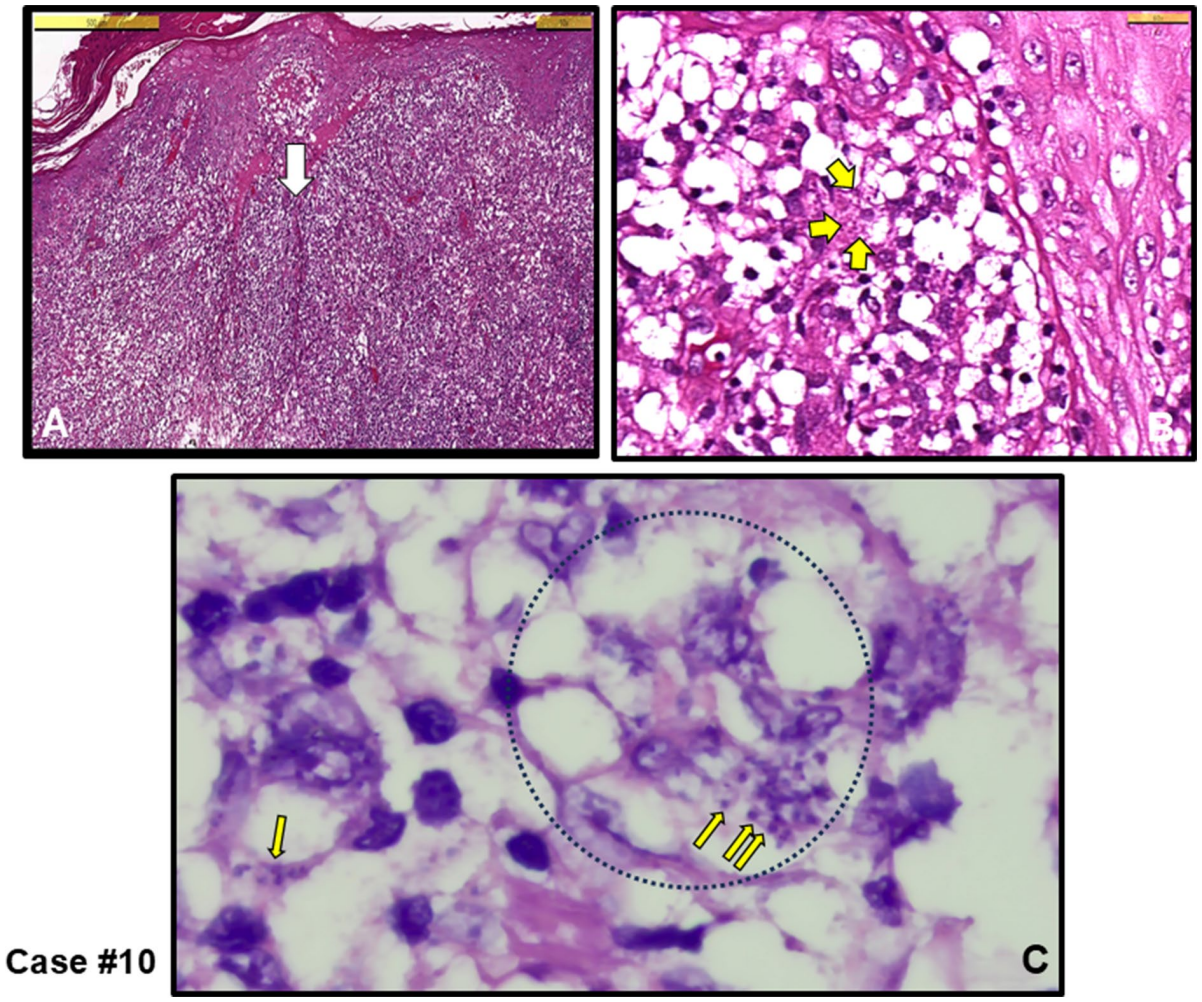
Case #10, a case with NGD initially clinically pre-diagnosed as skin adnexal tumor (SAT), and the lesion was completely excised. **(A)** Irregular acanthosis, spongiotic changes in the epidermis, and edema in the dermis with chronic inflammation rich with diffuse histiocytes (H&E stain, Scale bar 500 μm). **(B)** White arrow area in A. Yellow arrows show amastigotes (H&E stain, Scale bar 50 μm). **(C)** Oil magnified Giemsa stained sections. Thin yellow arrows show *Leishmania* amastigotes within giant histiocytes (example black dotted circle) (Giemsa stain, magnified with oil). *L. infantum* detected by PCR.

The other three cases exhibited mixed-type inflammation, characterized by a dense presence of histiocytes in the dermis (see cases #3, 11,12). Figure 3 provides an example of granuloma structures in the dermis, featuring cell debris with amastigotes present in the necrotizing areas (Figures 3A–C). Additionally, case #10, depicted in Figure 4, shows epidermal spongious changes and dermal edema (Figures 4A,B), with chronic infiltration of histiocytes filled with amastigotes (Figure 4C).

When necrotizing granulomatous dermatitis (NGD) features were observed microscopically, *Histoplasma capsulatum*, an intracellular fungus of similar size (2–4 μm) to the amastigote form of the *Leishmania* parasite, and *Mycobacterium tuberculosis* (Tbc), known to induce necrotizing granuloma, were included in the differential diagnosis. Therefore, histochemical stains such as PAS and GMS were employed to detect fungal hyphae and spores, while EZN stains were used to identify mycobacterial bacilli. In all four NGD, cases no specific causative agents for fungal or Tbc infections were detected after staining (data not shown).

Two out of seven cases (cases #8 and #12), representing 28.6%, were initially suspected of leishmaniasis and were directly diagnosed with CL upon pathological evaluation. In the remaining five cases, the amastigote form of the intracellular parasite could not be definitively identified. Therefore, the reports indicated that these cases were strongly suggestive of CL (MFSS of CL), and further microbiological evaluation was sought (cases # 3, 4, 5, 7 and 9).

Among the cases, five were submitted to the pathology laboratory with a pre-diagnosis of skin tumors, with no suspicion of an infectious agent (cases #1, 2, 6, 10 and 11). Notably, in four of these cases (cases #1, 2, 6 and 10), the lesions were completely excised. Three of these cases were directly diagnosed with CL upon identification of an amastigote (cases #1, 2 and 10 and Figure 4), while the remaining two were classified as having MFSS of CL (cases #6 and 11), prompting further microbiological evaluation. Taken together, after histopathological evaluation, amastigotes stained with H&E and Giemsa stains were clearly observed in the cytoplasm of histiocytes in 5 out of 12 skin biopsies, leading to a definitive diagnosis of CL (see example figures in Figures 1–4). In the other seven cases, although pathology reports indicated MFSS of CL, only descriptive diagnoses were provided, with recommendations for further microbiological confirmation.

### Identification of *Leishmania* species by PCR

**Figure 5:**
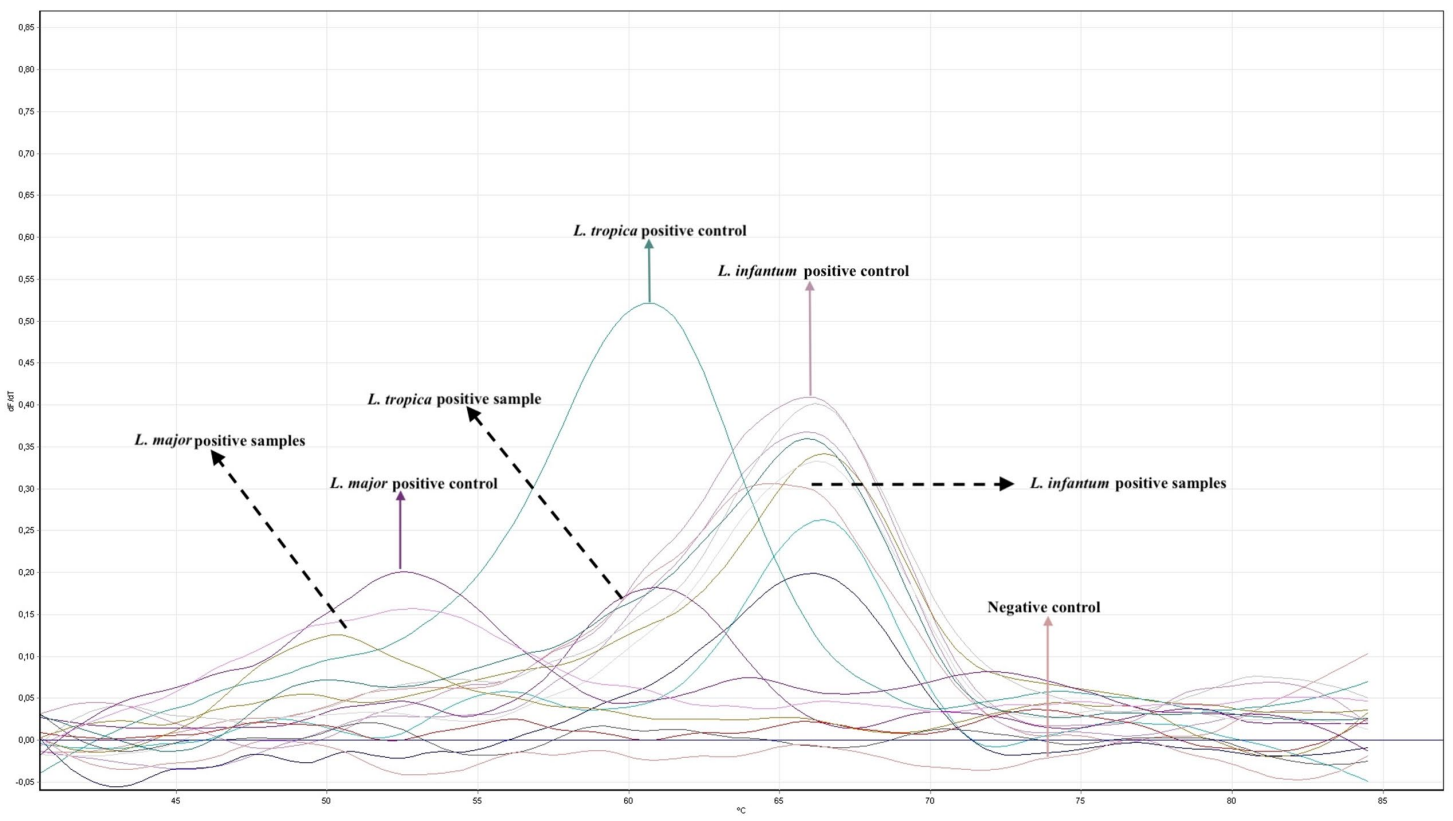
Quantitative real time PCR (RT-PCR) analysis using primers and probes specific for the ITS-1 region of *Leishmania*. The graph shows melting curve analyses of *L. major*, *L. tropica* and *L. infantum* reference strains and 12 patient isolates.

To investigate the effect of *Leishmania* species on the clinical pre-diagnosis, we performed DNA extraction and PCR from the paraffin-embedded skin biopsy block samples to establish the final diagnosis. Two types of PCR were conducted on the samples. *Leishmania infantum* was identified in 10 cases (83.3%) using both PCR methods, while *L. major* was detected in two cases (16.7%) using ITS-1 real-time PCR (Figure 5 and Table 1). Thus, altogether, 12 cases were proven to be CL by *Leishmania*-specific PCR.

## Discussion

The misdiagnosis of 12 patients with CL clearly indicates that CL can mimic the clinical manifestations of other diseases. Our findings, from a pathologists’ perspective, suggest that CL is not typically considered by physicians in previously non-endemic regions of Türkiye. Although both VL and CL have been notifiable diseases in Türkiye since the 1950s, the historical confinement of CL to specific areas has led to a lack of familiarity with CL among medical professionals. However, due to increased human migration at both national and international levels, CL is now emerging in previously non-endemic regions, including Europe. Additionally, histopathological diagnoses by pathologists were inconclusive in many cases, suggesting the need for adjunctive diagnostic methods, such as *Leishmania*-specific DNA detection techniques. Our analysis of *Leishmania-*specific DNA from paraffin-embedded tissue sections has revealed a complex epidemiological landscape of CL in Türkiye, including the unexpected detection of *L. infantum* causing CL. These findings highlight the evolving epidemiological and clinical challenges of leishmaniasis in Türkiye, which present significant diagnostic and treatment challenges for both physicians and pathologists.

Most cases of CL can heal spontaneously over time, often leaving behind scar tissue (3). However, in some cases-particularly in immunocompromised patients-there may be dissemination throughout the body, and if left untreated, persistent CL lesions may develop. Diagnosing CL can be challenging due to factors such as the variability in the duration of the self-healing process, the persistence of lesions, and the macroscopic appearance of the affected area. In our cases, ulcerated lesions were observed, some of which persisted for up to a year without healing. Although leishmaniasis was included in the clinical pre-diagnosis of seven patients, in five patients, the initial suspicion was confined to skin tumors, with CL not considered at all (Table 1). Notably, in two patients over the age of 70, the lesions were located on the face (cases #1 and #3). In these cases, skin tumors such as SCC and BCC were clinically considered due to the patients’ age, the sun-exposed location of the lesions, their macroscopic appearance, and the prolonged non-healing nature of the lesions.

Additionally, the differential diagnosis included other neoplastic skin conditions, such as skin adnexal tumors (SAT) in three cases, dermatofibroma in one case, and keratoacanthoma in one case, owing to the papulonodular lesions characteristic of CL. Previous studies from various regions, including non-endemic areas like Northern Europe, have repeatedly reported that CL can be misdiagnosed as skin cancers and other conditions such as epithelial neoplasms, follicular cysts, atypical mycobacteriosis, sarcoidosis and lymphoma (3, 33). Importantly, such misdiagnoses can lead to radical surgical interventions (as seen in our cases #1, 2, 6 and 10) and/or to the administration of inadequate treatments, such as corticosteroids, which further complicates histological diagnosis by pathologists.

Distinguishing between CL and skin tumors can be challenging due to several factors. *Leishmania* parasites replicate within macrophages, monocytes, and dendritic cells (34). Histologically, these lesions are characterized by aggregates of macrophages containing numerous intracellular parasites, accompanied by an intensified inflammatory response, which is attributed to the high antigenic density of the parasites (35, 36). The host’s ability to control the parasite is mediated by the activation of IFN-γ, an intracellular regulatory cytokine (34, 36, 37). The primary source of IFN-γ is CD4+ T cells, which are stimulated by macrophages, followed by CD8+ T and NK cells (34). If the parasite load is excessively high or if an adequate IFN-γ response is not developed, the disease may not be self-limiting (38). Consequently, extensive and/or persistent lesions can occur (34). This exaggerated inflammatory response leads to a significant tissue damage and loss (34, 36, 37), resulting in the development of ulcers and papulonodular lesions in the skin and subcutaneous tissue (21, 35). Macrophages, in conjunction with T lymphocytes, play a crucial role in controlling the growth of cancerous cells during the early stages of carcinogenesis. However, chronic inflammation, if left untreated, can contribute to tumor development and progression, often mediated by macrophages. Therefore, it is plausible that *Leishmania* parasites, which reside intracellularly and induce chronic inflammation, may create a predisposing environment for tumor formation (3, 39, 40).

Chronic granulomatous inflammation is a morphological pattern observed in a variety of diseases, including microbial infections caused by bacterial and fungal agents such as *M. tuberculosis* and *H. capsulatum*, respectively, as well as in a range of etiologically distinct conditions like sarcoidosis and rheumatoid arthritis (41–43). In our cases, necrotic granulomatous inflammation, which resulted in the formation of large lesions and was clinically misdiagnosed as skin tumors, was observed in infections caused by *L. infantum* (cases #1, 2, 6 and 10). While histiocytic inflammation is not only specific to leishmaniasis, the pathognomonic feature of the disease is the identification of amastigotes within histiocytes or macrophages. When leishmaniasis is clinically suspected, it facilitates the pathologist’s diagnosis. The detection of the intracellular parasite is sometimes possible even with H&E staining. In cases where amastigotes are not clearly visible in H&E-stained sections, Giemsa staining can assist in the diagnosis (Figures 1–4).

Another challenge with diagnosing CL is that histopathological findings can vary depending on the *Leishmania* species. In the current study, *L. infantum* was identified in 10 cases using ITS-1 real-time PCR. However, since the ITS-1 region does not effectively differentiate between *L. infantum* and *L. donovani*, and given that *L. donovani* as a rare cause of CL in Türkiye (29), cpb PCR was performed to distinguish between these two species in the 10 samples identified as *L. infantum* by real-time ITS-1 PCR. All 10 samples were confirmed as *L. infantum* by cbp PCR (Figure 5). *L. infantum* is widely recognized as the causative agent of zoonotic leishmaniasis, primarily affecting dogs (canine leishmaniasis, CanL) in the Mediterranean Basin, and it is the most prevalent species responsible for human VL (11). In contrast, *L. tropica* and *L. major* are typically more dominant in cases of CL. However, in recent years, due to extensive human migration and prolonged tourism in endemic regions, *L. infantum* species has become increasingly prevalent in CL cases (11, 44).

The evolving epidemiology of human and canine leishmaniasis, particularly in areas where sand flies are prevalent, introduces several complexities in re-emergence scenarios. Although this study was conducted in a single center in Istanbul, patients’ anamneses suggest that some individuals may have acquired the infection in other cities within Türkiye (such as Gaziantep, Adana) or abroad (including Brazil, Lebanon, and Kuwait), all of which are known endemic regions for CL. Additionally, it is well-documented that many Istanbul residents maintain strong connections with their rural origins, particulaly during the summer holidays, which coincide with the peak season for infection. Notification of VL and CL is mandatory in Türkiye, and some of the CL patients included in this study were from endemic regions where autochthonous CL cases have been previously reported, particularly in the Adana and Gaziantep provinces (25, 45). Four Old World *Leishmania* species are present in Türkiye, one of which, *L. infantum*, causes both human and canine VL and CL in endemic areas (46). The strains of *L. infantum* isolated from CL patients and sand flies in the Adana province, as well as from VL and CanL patients in various parts of Türkiye, have been identified as *L. infantum* MON-309 and *L. infantum* MON-1, respectively, with the latter being a common zymodeme in Mediterranean Basin countries (47). The difference in zymodeme suggests genetic heterogeneity (48). Sand fly surveys in VL/CL/CanL endemic areas where *L. infantum* is the causative agent have identified *Phlebotomus tobbi* as the proven vector species and *P. major* s.l. as a probable vector species (49).

Notably, dogs are the most important reservoirs of *L. infantum* for VL in Türkiye, with higher seroprevalence rates than those observed in human cases in endemic areas (31). Studies on CanL have also shown moderate seroprevalence in Istanbul (50) and high seroprevalence in regions such as Adana, Gaziantep, and Bodrum (31, 51). Of the 12 described cases in this study, 11 presented with a singular lesion. Only in case #12 did nodular lesions initially appear on the forehead and later spread to the extremities. In this atypical case #12 (Figure 3) caused by *L. infantum*, the diffuse cutaneous lesions may suggest either repeated sand fly bites or a disseminated infection due to immune deficiency (16, 19). We recommend that all CL patients undergo periodically follow-up even after treatment.

In summary, leishmaniasis is a serious global health threat that is becoming more prevalent due to wars and mass human migrations. This disease poses a particular risk to countries like Türkiye, which is situated in a geographical transition zone (11, 52). It is well-recognized that Türkiye and Europe will face an increasing number of leishmaniasis cases, a disease that is becoming more significant in the medical field (53). We recommend that leishmaniasis should routinely be considered in the differential diagnosis of skin lesions. This is especially important in cases of granulomatous skin inflammation, with or without necrosis, and/or chronic inflammation dominated by histiocytes. A leishmaniasis-focused diagnostic approach should be employed, with careful examination for the intracellular form of the parasite. PCR confirmation is advised when the intracellular form of the parasite is not visible microscopically, but morphological findings raise suspicious of CL. Although CL can be diagnosed histopathologically, it is essential to identify the specific type of leishmaniasis, as different *Leishmania* species may require different drug treatments (21).

## Data Availability

The raw data supporting the conclusions of this article will be made available by the authors, without undue reservation.
